# Naphthylacetic Acid and Tea Polyphenol Application Promote Biomass and Lipid Production of Nervonic Acid-Producing Microalgae

**DOI:** 10.3389/fpls.2018.00506

**Published:** 2018-04-17

**Authors:** Feng Xu, Yong Fan, Fuhong Miao, Guang-Rong Hu, Juan Sun, Guofeng Yang, Fu-Li Li

**Affiliations:** ^1^Forage Research and Development Center for Arable Region, Qingdao Agricultural University, Qingdao, China; ^2^Shandong Provincial Key Laboratory of Synthetic Biology, Qingdao Institute of Bioenergy and Bioprocess Technology, Chinese Academy of Sciences, Qingdao, China

**Keywords:** *Mychonastes afer*, photosynthetic efficiency, reactive oxygen species, photosynthetic system II, CP43, bioactive additive

## Abstract

*Mychonastes afer* HSO-3-1 is a potential producer of nervonic acid, which could be accumulated to 2–3% of dry cell weight. Improving the productivity of nervonic acid is critical to promote the commercialization of this product. In this study, 1-naphthylacetic acid (NAA) and tea polyphenol (TP) were selected as bioactive additives to stimulate the growth of *M. afer*. Supplementing NAA in the early growth stage and TP in the middle and late growth stage led to improved lipid accumulation in *M. afer*. The cultures supplemented with TP at the late growth stage maintained higher photosynthetic efficiency than the control groups without TP. Furthermore, the intracellular reactive oxygen species (ROS) accumulations in *M. afer* supplemented with 500 mg/L of TP was 63% lower than the control group. A linear relationship (*R*^2^= 0.899) between the values of Fv/Fm and ROS accumulation was established. We hypothesize supplement of bioactive additives at different growth stage could promote the cell growth rate and nervonic acid productivity of *M. afer* by retrieving intracellular ROS level. Further analysis of photosynthetic system II (PSII) protein in *M. afer* cultured in presence of NAA and TP indicated the levels of D1 and D2 proteins, the core skeleton proteins of PSII, showed 33.3 and 25.6% higher than the control group. CP43 protein, a critical module in PSII repair cycle, decreased significantly. These implied that TP possesses the function of slowing down the damage of PSII by scavenging excess intracellular ROS.

## Introduction

As a promising feed-stock of biodiesel or jet fuel, microalgae have received extensive attention in recent years due to its ability of producing substantial amounts of triacylglycerols (TAGs) under stress conditions (Hu et al., 2008). The concept of using microalgae as an alternative and renewable source of lipid-rich biomass feed-stock for biodiesel has been explored over the past few decades, while technical and economic barriers still limit the development of the whole industry chain (Williams and Laurens, 2010). Outstanding strains with high lipid yield and effective culture technology are main challenges for commercialization of microalgal biodiesel (Chisti, 2007).

*Mychonastes afer* HSO-3-1 is a promising candidate for biodiesel production, because of its high lipid content (50% dry cell weight) and fast growth rate. Furthermore, nervonic acid (NA, C24:1 Δ15, *cis-*tetracos-15-enoic acid, ω-9) could account for more than 5% of the neutral fatty acids in *M. afer.* NA, a very long chain monounsaturated fatty acid (VLMFA), is essential for brain development (Sandhir et al., 1998; Yuan et al., 2011; Fan et al., 2018b). It could be used to treat genetic disorders of the lipid metabolism, such as Zellweger syndrome or adrenoleukodystrophy (Coupland and Langley, 1993; Fan et al., 2018a). An impairment in the provision of NA in demyelinating diseases (like multiple sclerosis) suggests that a diet rich with NA could improve the treatment (Sargent et al., 1994).

In order to increase the productivity of NA in *M. afer*, optimization of the culture conditions should be developed firstly. Phytohormones are found not only in higher plants but also in algae, and the biological activities of hormones in algae are similar to the functions of hormones in higher plant (Lu and Xu, 2015). Exogenous IAA (0.1–10 μM) increased *Chlamydomonas reinhardtii* biomass production by 54–69% (Park et al., 2013). The supplement of IAA (0.1 μM) or 1-naphthylacetic acid (NAA) (1 μM) induced a significant increase in cell number by 53 and 24% in *Chlorella vulgaris*, and it would increase the amount of monosaccharides, photosynthetic pigments, and soluble enzymes in *C. vulgaris* (Piotrowska-Niczyporuk and Bajguz, 2014).

Compound additives will be a trend in the cultivation process, and the combination of additives needs to be analyzed and tried unceasingly. According to our previous research, intracellular reactive oxygen species (ROS) level is another key factor that influences the growth of microalgae. Plants maintain complex systems of overlapping antioxidants to balance the oxidative state *in vivo* (Munne-Bosch, 2005), while microalgal antioxidant system may not deal with the over-oxidation stress efficiently due to its simple cell structure (Cavas and Yurdakoc, 2005). Reactive oxygen species can function as signaling molecules that control the cellular basal metabolism such as induced cell re-programming toward programmed cell death or many other developmental processes. Unbalanced oxidative state will cause abiotic stress responses in plants, and excess ROS accumulation is toxic to cells (Mittler, 2002). Antioxidants can prevent cells from ROS accumulation by scavenging the excessive ROS (Wolf, 2005). In addition, algal antioxidant system plays crucial roles in stress tolerance, like heavy metal, salinity and heat stress (Geider et al., 1993; Pinto et al., 2003; Liu and Pang, 2010). In our previous work, varieties of potential candidate additives were selected from phytohormones and antioxidants, like NAA, indole-3-acetic acid, gibberellic acid, kinetin, abscisic acid, tocopherol, epigallocatechin-3-gallate (EGCG), and tea polyphenol (TP) (Frei and Higdon, 2003). In consideration of solubility, stability, and photo-sensitivity, one phytohormone (NAA) and two antioxidants (EGCG and TP) were finally selected for further investigation of the effects on the growth and lipid accumulation of *M. afer*. We hypothesize that the supplement of bioactive additives at different growth stages could promote the cell growth rate and nervonic acid productivity of *M. afer*. Orthogonal experiments were used to analyze the additive doses, and it was further confirmed by single factor experiment analysis. We found that the supplement of these additives at different growth stages led to different effects on microalgal growth. Interestingly, supplement of TP increases the tolerance to ROS in *M. afer* at later growth stage. Photosynthetic efficiency of the cells cultured in presence of NAA and TP was analyzed.

## Materials and Methods

### Strains and Culture Conditions

*Mychonastes afer* was stored in the China General Microbiological Culture Collection Center with the identifying code CGMCC No. 4654. It was cultured in a column photobioreactor (20 cm high, 4 cm diameter, 100 mL culture volume) under continuous illumination at light intensity of 120 μmol photons m^-2^⋅s^-1^. Culture mixing and aerating was provided by aeration with filter-sterilized air containing 2% CO_2_ (Yuan et al., 2017). The initial OD_750_ of algal culture was 1.0. All strains were cultured at room temperature (25 ± 2°C) for 12 days. The cells were grown in a modified BG-11 medium with 0.374 g/L NaNO_3_ (Li et al., 2011). The other compositions of the BG-11 medium are as follows: 0.03 g/L K_2_HPO_4_, 0.075 g/L MgSO_4_⋅7H_2_O, 0.036 g/L CaCl_2_⋅2H_2_O, 0.006 g/L citric acid, 0.006 g/L ferric ammonium citrate, 0.001 g/L EDTA, 0.02 g/L Na_2_CO_3._ And 1 ml of trace metal solution per liter medium, which content: 2.86 g/L H_3_BO_3_, 1.81 g/L MnCl_2_⋅4H_2_O, 0.222 g/L ZnSO_4_⋅7H_2_O, 0.39 g/L NaMoO_4_⋅5H_2_O, 0.079 g/L CuSO_4_⋅5H_2_O, 0.0494 g/L Co(NO_3_)_2_⋅6H_2_O. The culture medium was sterilized in an autoclave at 121°C for 20 min. TP (Food grade, Xitang Biological Technology, Co., Ltd., China), EGCG and NAA (TCI Development, Co., Ltd., Shanghai, China) were sterilized by filtration.

In the first step, orthogonal experiments were carried out with four levels as follows: 0 mg/L (A_1_), 0.5 mg/L (A_2_), 1.5 mg/L (A_3_), and 5 mg/L (A_4_) for NAA dosage, 0 mg/L (B_1_), 0.5 mg/L EGCG (B_2_), 2 mg/L TP (B_3_), and 20 mg/L TP (B_4_) for antioxidants dosage. And 120 μmol photons m^-2^⋅s^-1^ of light intensity was used as low-light level (LL, C_1_/C_2_), 400 μmol photons m^-2^⋅s^-1^ was used as high-light level (HL, C_3_/C_4_). Three-factors-four-levels experiments were conducted by the orthogonal list *L*_16_ (4^5^). NAA was added at the early growth stage when the OD_750_ of algal culture was about 3.0 at day 2. EGCG and TP were added at the middle growth stage when the OD_750_ of algal culture was about 6.0 at day 6. Experimental scheme and results were shown in Supplementary Table 1. All data were analyzed by SPSS Statistics (Version 19, IBM, Armonk, NY, United States).

To confirm the effects of the candidate factors selected by orthogonal experiments, single factor experimental scheme was conducted with a wider gradient of the additive concentration. A new range of NAA concentrations (0, 0.1, and 10 mg/L) and TP concentrations (0, 50, and 500 mg/L) were chosen. Nine experimental groups were as follows: CT, 0.1 NAA, 10 NAA, 50 TP, 500 TP, 0.1 NAA+50 TP, 0.1 NAA+500 TP, 10 NAA+50 TP, and 10 NAA+500 TP. An additive combination of NAA (0.1 mg/L) and TP (500 mg/L) in the medium was expressed as 0.1 NAA+500 TP. CT was the control group without additives.

### Algal Biomass and Chlorophyll Fluorescence Measurements

Algal samples (10 mL) were filtered through the pre-weighed filter paper (0.22 mm, Whatman International, Ltd., Maidstone, United Kingdom) and dried at 105°C for 8 h. The difference between the final weight and initial weight of the filter paper was the dry weight of the samples.

Algal cells suspension was transferred to the 96-well plates and analyzed by Imaging-PAM Chlorophyll Fluorometer (Walz, Effeltrich, Germany). The chlorophyll fluorescence was measured and calculated, the data of photosynthetic efficiency were determined, including the data of Fv/Fm, non-photochemical quenching (NPQ) and quantum yield of photochemical energy conversion in PSII (YII) (Klughammer and Schreiber, 2008; Hu et al., 2013).

### Lipid Extraction, Quantification, and Composition Analysis

Nile red staining was carried out for measurement of neutral lipids in microalgae (Chen et al., 2009). Freeze-dried biomass was further used for total lipid extraction (Bigogno et al., 2002). Following procedures: 30 mg dry algae powder was resuspended with the chloroform-methanol (4 vs. 2 ml) to extract the lipid by shaking with a speed of 200 rpm at 37°C for 2 h. After centrifuging at 3200 *g* for 10 min, the supernatant was transferred to a new tube and mixed with 2 ml methanol and 3.6 ml 5% NaCl. The remaining lipid was dissolved in chloroform phase by centrifuging at 3200 *g* for 5 min, then the chloroform phase was transferred into pre-weight vials. The residuals were weighed after removing chloroform by nitrogen evaporator.

Total lipid composition was analyzed after methyl esterification processing by reacting with 2% H_2_SO_4_ in methanol at 85°C for 3 h. Gas chromatography analysis was carried out with a GC system (7890A, Agilent Technologies, Inc., Santa Clara, CA, United States). HP-5 (30 m × 320 μm × 0.25 μm, Agilent, United States) was selected as the chromatographic column. Gas heating process was as follows: rise the column temperature to 190°C with a heat rate of 10°C/min, and maintain 1 min. Then rise the temperature to 207°C with a heat rate of 0.8°C/min, and maintain 1 min. N_2_ was used as carrying gas with a speed (28.5 mL/min) and split ratio (10:1, *v/v*). Heptadecanoic acid (C17:0, 3 mg/mL) was used as an internal standard.

### Intracellular ROS Level, Lipid Peroxidation, and ROS Scavenging Enzyme (Catalase) Activity Analysis

Intracellular ROS analysis was performed by using a Reactive Oxygen Species Assay Kit (Beijing Solarbio Science & Technology, Co., Ltd., China; code: CA1410-100T) according to the manufacturer’s instructions. This kit used 2,7-Dichlorodi-hydrofluorescein diacetate (DCFH-DA) as probe. This non-fluorescence probe could be oxidized by intracellular ROS, including hydrogen peroxide (H_2_O_2_) and hydroxyl radicals (OH∙), to the fluorescence probe of DCF. This fluorescence was most sensitive to the level of H_2_O_2_ and could be used to compare the levels of this ROS in different conditions of the cells. Culture samples of *M. afer* were collected and adjusted to a final cell concentration of 1 × 10^7^ cells per milliliter with culture medium which collected from the culture system. Then cells were incubated with 1 μM DCFH-DA for 20 min under culture condition (same light intensity and temperature). DCF fluorescence was measured with 488 nm excitation wavelength and 525 nm emission wavelength by a multi-mode microplate reader (SynergyTM HT, BioTek, Winooski, VT, United States).

Malondialdehyde (MDA, the by product of lipid peroxidation) content and Catalase (CAT) activities were measured according to Shi et al. (2009). MDA and CAT kits were purchased from Nanjing Bioengineering Institute, China.

### Thylakoid Membrane Preparation and Protein Level Analysis

Microalgae cells were harvested by centrifugation at 4000 *g* for 10 min and resuspended in 10 ml of Hepes-OH buffer (20 mM, pH 7.5). After centrifuged at 3200 *g* for 5 min, the pellets were suspended with 5 ml of extraction buffer (300 mM sorbitol; 50 mM Hepes-OH, pH 7.5; 2 mM Na_2_EDTA, pH 8.0; 1 mM MgCl_2_⋅6H_2_O and 1% BSA). The microalgae cells were homogenized by grinding in liquid nitrogen and the extraction were performed in triplicates. All subsequent steps were carried out on ice. The supernatant was collected in centrifuge tube by centrifugation at 4000 *g* for 10 min. Following centrifugation at 13000 *g* for 30 min with the collected supernatant, the precipitated thylakoid membranes were resuspended in extraction buffer.

For the qualification of chlorophyll and protein content in thylakoid, thylakoid membrane was soaked with 80% acetone at 60°C for 2 h, then separate the chlorophyll and protein by centrifugation at 13000 *g* for 15 min. Chlorophyll content in thylakoid was determined by the method published (Wellburn, 1994). And the total protein content was determined by BCA Protein Assay Kit (by protein reduction chromogenic reaction) following the manufacturer’s instructions (Beijing Solarbio Science & Technology, Co., Ltd., China).

Sodium dodecyl sulfate polyacrylamide gel electrophoresis (SDS-PAGE) was performed according to the protocol (Ikeuchi and Inoue, 1988), and thylakoid membrane proteins were used as testing samples. For semi-quantification analysis, the gray scale of each band was measured by Image J software. Target bands which had significant differences were excised and decolorized using decolor-buffer (100 mM NH_4_CO_3_/30% acetonitrile) at 4°C for 2 h. This step was repeated two times until the spot gels were totally clean. After centrifugation at 13800 *g* for 5 min, the pellets of gels were lyophilized and then transferred in 5 μl Trypsin solution (12.5 ng/μl in 50 mM NH_4_HCO_3_) at 4°C for 60 min, followed by adding 20 μl 50 mM NH_4_HCO_3_ at 37°C for 20 h. The enzymatic hydrolysate was transferred to another tube, and the supernatant was used for LC–MS/MS analysis.

Samples were analyzed on a Surveyer plus a LC-LTQ XL (Thermo Fisher Scientific, Inc., Waltham, MA, United States). The electrospray voltage of 2.2 kV was carried out with the ion transfer tube temperature at 220°C. Digested peptides were analyzed by using data-dependent acquisition of a MS scan (600–2000 m/z), and then MS/MS scans were performed for the three most abundant ions in each MS scan. Normalized collision energy for MS/MS was set to 35% with an isolation width of 1.5 amu. From raw files, MS/MS spectra were exported to individual files in data format according to the following setup: peptide mass range, 350–5000 Da; minimal total ion intensity threshold, 1000; minimal number of fragment ions, 15; precursor mass tolerance, 1.4 amu; group scan, 1; minimum group count, 1.

Extracted MS/MS spectra were converted to a Mascot genetic format file (mgf) and searched against a *C. reinhardtii* database [from the National Center for Biotechnology Information (NCBI), updated November 17, 2017, containing 33,220 entries] or Swiss_Prot (updated November 17, 2017, containing 560,836 entries) by Mascot (Version 2.4, Matrix Science, Ltd., London, United Kingdom).

For western-blot analysis, thylakoid membrane proteins of *M. afer* were used as total protein samples. Primary antibodies [D1 protein of photosynthetic system II (PSII), C-terminal, rabbit antibody, code: AS05_084] were purchased from Agrisera, Co., Ltd., Sweden. Western-blotting was performed by probing with specific antibodies after electroblotting onto nitrocellulose membranes (GE Healthcare, Co., Ltd., Chicago, IL, United States) (Liu et al., 2012b). Primary antibody was diluted 10000-fold (antibody against D1), and signals from horseradish peroxidase-conjugated goat anti-rabbit IgG (H+L) were visualized using Clarity Western ECL substrate (MDBio, Inc., Qingdao, China) and the blotting-spots were analyzed using ImageJ software (National Institutes of Health, United States) (Liu et al., 2012a).

### Statistical Analysis

All the experiments were repeated three times. Unless otherwise stated, all data were expressed as mean standard deviation (SD). Statistical significance and determination coefficients of the values obtained from each experiment was evaluated by variance (ANOVA) using the software SPSS (version 19.0, IBM, Chicago, IL, United States). Significant differences were considered when *p* < 0.05.

## Results

### Growth and Lipid Content of *M. afer* After NAA and Antioxidants Supplement

**FIGURE 1:**
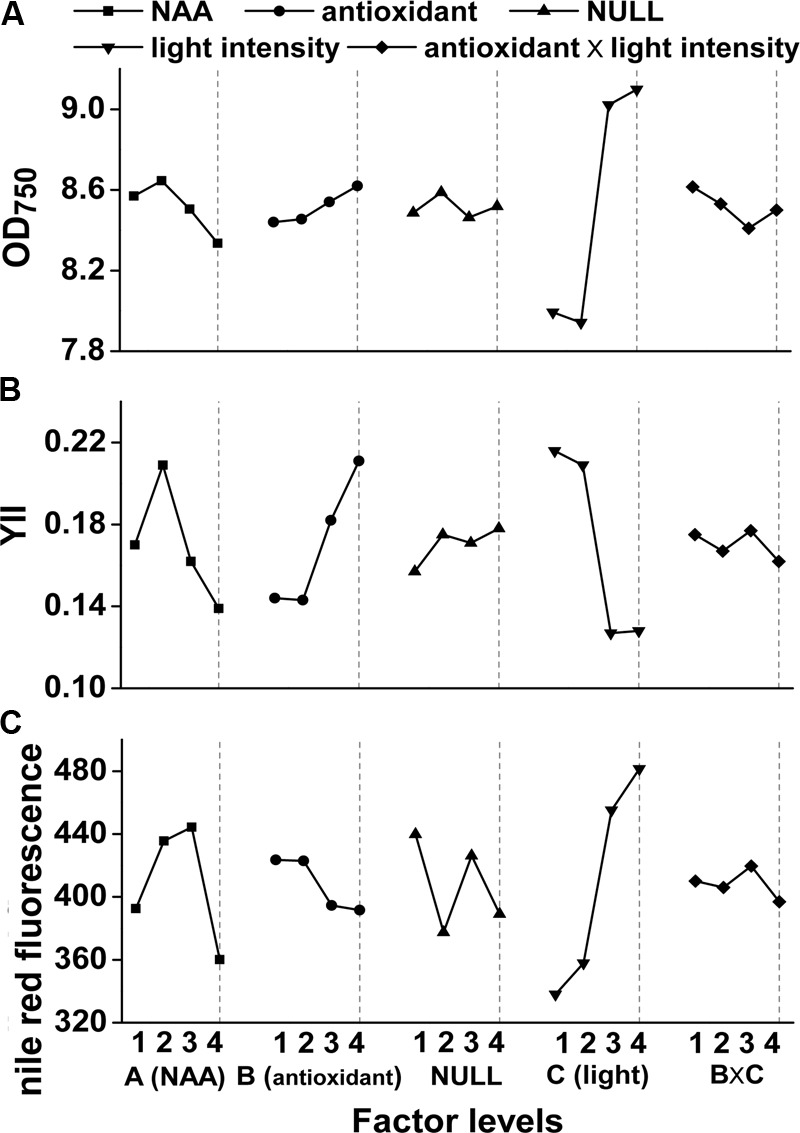
Orthogonal effect curves of NAA, antioxidants, and light intensity on growth (OD_750_), photosynthetic efficiency (YII), and lipid accumulation of *Mychonastes afer*. NAA **(A)**, antioxidant **(B)**, light intensity **(C)** and the interactions between the light intensity and antioxidants (B × C) were the factors investigated here with four levels, and NULL is for the error assessment. The four levels of these factors were as follows: 0 mg/L (A_1_), 0.5 mg/L (A_2_), 1.5 mg/L (A_3_), and 5 mg/L (A_4_) for NAA dosage, 0 mg/L (B_1_), 0.5 mg/L EGCG (B_2_), 2 mg/L TP (B_3_), and 20 mg/L TP (B_4_) for antioxidants dosage. And 120 μmol photons m^-2^⋅s^-1^ of light intensity was used as low-light level (LL, C_1_/C_2_), 400 μmol photons m^-2^⋅s^-1^ was used as high-light level (HL, C_3_/C_4_).

Experimental scheme and results of the orthogonal experiments were shown in Supplementary Table 1. Light intensity, NAA and antioxidants all had significant (*p* < 0.05) influence on *M. afer* photosynthetic efficiency (Supplementary Table 3), while the growth was only significantly (*p =* 0.001) affected by light intensity (Supplementary Table 2). No significant effect on lipid content was observed from all of these factors (Supplementary Table 4). Microalgal photosynthetic efficiencies were significantly influenced by NAA (*p* = 0.046) and antioxidants (*p* = 0.033), while these effects were not reflected by the growth of *M. afer* (**Figure 1**).

**FIGURE 2:**
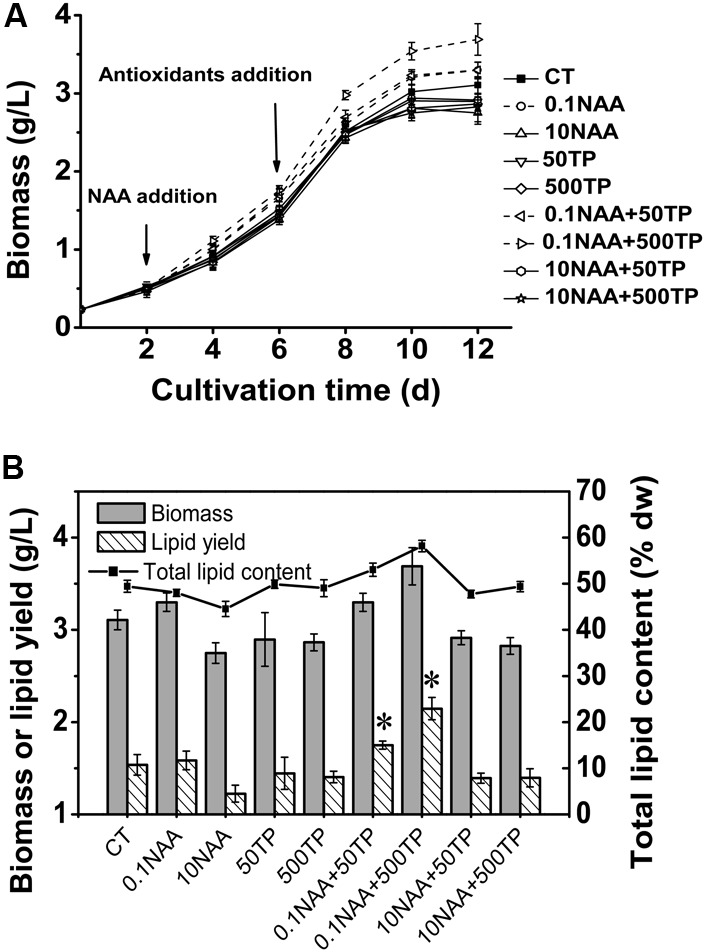
Growth and lipid content of *M. afer* supplemented with NAA and TP. **(A)** Growth curves of *M. afer* that grew under different treatment of additive combinations. CT is the control group without additives. NAA and TP were added at day 2 and day 6, respectively. **(B)** Final algal biomass (dry weight), lipid content (% dry weight), and lipid yield of *M. afer* under different culture conditions. Values are means ± SE (*n* = 3). Asterisks indicate statistically significant differences compared with the control group (^∗^*p* < 0.05; ANOVA).

Based on the relationship between photosynthetic efficiency of *M. afer* and additives, new experimental scheme was conducted with a wider gradient of the additive concentration to further verify the orthogonal experiment results. The algal growth rate increased with an addition of 0.1 mg/L NAA on the 2nd day of cultivation, and this promotion was amplified when TP was added on the 6th day of cultivation. After the supplement of NAA (0.1 mg/L) and TP (500 mg/L) in the medium, the maximum algal biomass was 3.69 g/L, which was 18.76% higher (*p* = 0.002) than control (**Figure 2A**).

Total lipid content of *M. afer* was increased with the supplementary of 0.1 mg/L NAA and TP. The maximum algal total lipid content and lipid yield was 17.6 and 39.6% higher than control without additives, respectively. It appeared in the experimental group with the supplement of NAA (0.1 mg/L) and TP (500 mg/L) in the medium (**Figure 2B**). However, no significant difference of fatty acids composition was observed under different culture conditions, according to the GC analysis data (Supplementary Table 5).

### Effects of NAA and TP on Photosynthetic Efficiency of *M. afer*

**FIGURE 3:**
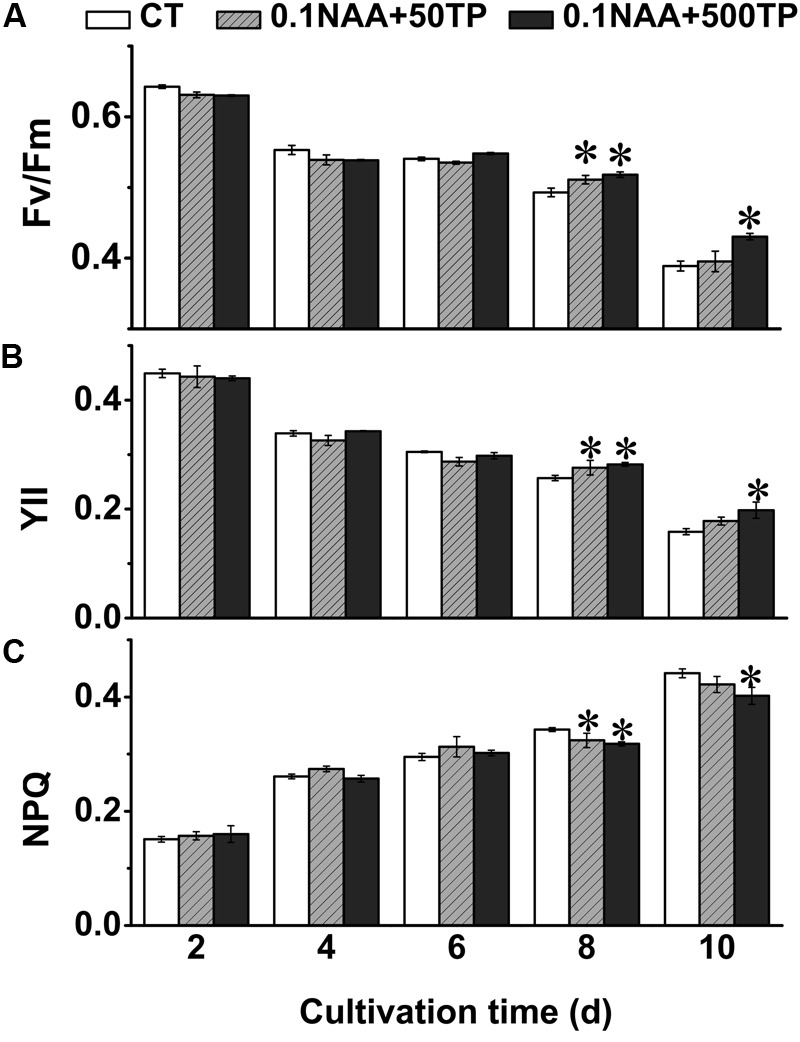
Effects of NAA and TP on photosynthetic efficiency of *M. afer.* Photosynthetic parameters Fv/Fm **(A)**, YII **(B)**, and NPQ **(C)** were detected. NAA and TP were added at day 2 and day 6, respectively. Values are means ± SE (*n* = 3). Asterisks indicate statistically significant differences compared with the control group (^∗^*p* < 0.05; ANOVA).

Microalgae utilize light energy to maintain the energy requirements for life activities and growth. In order to elucidate the mechanism for the higher growth rate and lipid content of *M. afer* after adding NAA and TP, the photosynthetic efficiency of *M. afer* under different culture conditions were measured. Fv/Fm was the potential maximum photosynthetic activity of the photosynthetic organisms. Fv/Fm could also reflect the activity of the PSII complex. YII stands for the actual quantum yield (actual photosynthetic efficiency) of PSII in any light state and can reflect the activity of linear electron transport (Klughammer and Schreiber, 2008). NPQ reflects the ability of photosynthetic organisms to dissipate excess light to heat. The Fv/Fm and YII of *M. afer* decreased in the later stage of culture, while some groups which added TP at day 6 showed higher Fv/Fm than control group (*p* = 0.002). Consequently, the NPQ of *M. afer* increased in the later stage of culture, while the groups added with TP would lead a lower NPQ, indicating a lesser light energy dissipation in the cells with TP supplement (**Figure 3**). These results suggested that TP could maintain higher photosynthesis efficiency of *M. afer* at later growth stage compared with the control group.

### Intracellular Reactive Oxygen Species (ROS), Lipid Peroxidation, and ROS Scavenging Enzyme (CAT) Activity Analysis

**FIGURE 4:**
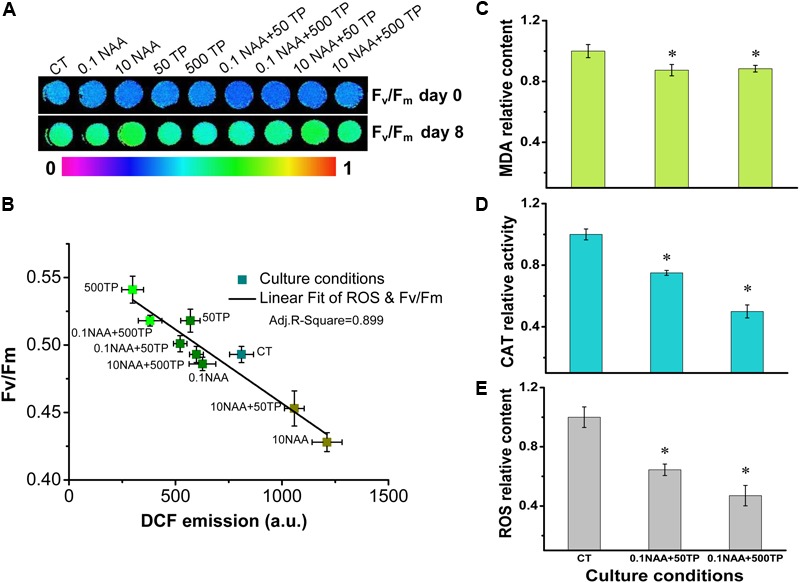
Intracellular ROS analysis of *M. afer* and their relationship with the Fv/Fm values under different culture conditions. **(A)** False-color images showed Fv/Fm variations of *M. afer* under different culture conditions. Algal cells suspension was added to the 96-well plates. After 10 min of dark adaptation, Fv/Fm was determined following a saturating pulse of light, and chlorophyll fluorescence images of algal cells suspension were obtained at this time point. **(B)** Linear fit of ROS and Fv/Fm. DCF fluorescence was used to semi-quantitative analyze the accumulation of H_2_O_2_ in *M. afer*. Quantification analysis of intracellular ROS level **(C)**, MDA content **(D)**, and CAT activity **(E)** of *M. afer* under different culture conditions. *M. afer* cells were harvested and analyzed immediately at day 8. Values are means ± SE (*n* = 3). Asterisks indicate statistically significant differences compared with the control group (^∗^*p* < 0.05; ANOVA).

At the late growth stage, algae grew under stress conditions due to deplete of nutrition in medium, which in general led to decreased energy converting efficiency (Kolber et al., 1988). Excess light energy would lead to sustained increases in intracellular ROS level, which would have detrimental effects on algal growth (Choo et al., 2004). The observation that cells of *M. afer* could maintain high photosynthetic efficiency at later growth stage after TP supplement, implying that TP might regulate the photosynthesis efficiency of *M. afer* by alleviating its intracellular ROS. We selected day 8 as the time point to analyze the relationship between photosynthetic efficiency of *M. afer* and its intracellular ROS level, because the photosynthetic efficiency of *M. afer* showed the most significant difference between the control group and the cells cultured with 0.1 mg/L NAA and 500 mg/L TP at this time point (*p* = 0.0004). A linear relationship was observed between Fv/Fm values and intracellular ROS levels, which suggested that TP could promote the photosynthesis efficiency of *M. afer* by alleviating its intracellular ROS at later growth stage (**Figures 4A,B**). Base on the method, the species of these reactive oxygen were mainly H_2_O_2_. The cells of two experimental groups (0.1 NAA+50 TP and 0.1 NAA+500 TP) which had the highest lipid yield were harvested for further MDA content and CAT activities analysis. MDA is the by product of lipid peroxidation. MDA content was analyzed as the indicator for lipid peroxidation, and the excess ROS accumulation in cells can be represented by their lipid peroxidation (Zhang et al., 2013). CAT is a key enzyme which is located in peroxisomes and mitochondria in ROS scavenging system (Kato et al., 1997). CAT became more abundant in the later stage of the cell culture, and higher CAT activity reflected a later stage of the cell culture. Over-expression of CAT in cytosolic or mitochondrial compartment protects cells against oxidant injury (Bai et al., 1999). Intracellular ROS level (DCF fluorescence) of *M. afer* cultured with NAA (0.1 mg/L) and TP (500 mg/L) were significantly lower than the control group, which decreased 53% (**Figure 4C**). Correspondingly, MDA content and CAT activity of *M. afer* cultured with NAA (0.1 mg/L) and TP (500 mg/L) were about 88.4 and 50% compared with control group, respectively (**Figures 4D,E**). The lower intracellular ROS level and MDA content indicated that the cells of *M. afer* cultured with TP supplement were under lower oxidant stress than the control group. Meanwhile, a lower CAT activity was enough for excessive ROS neutralization in TP supplemented cultures.

### Western-Blot Analysis of Photosynthetic Proteins

**FIGURE 5:**
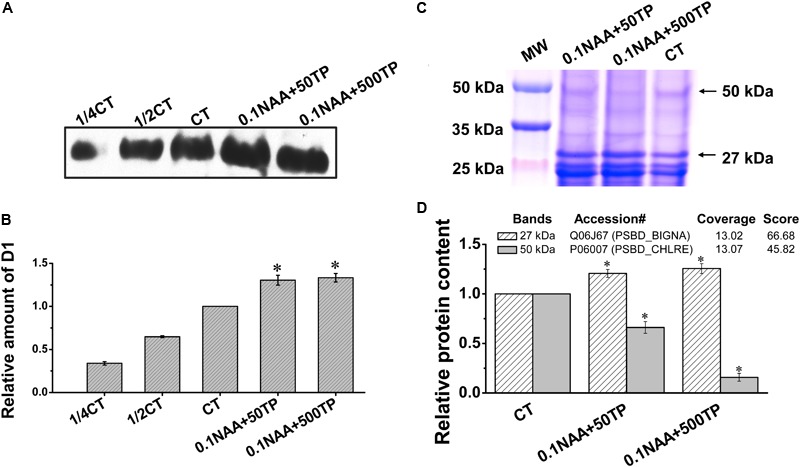
Quantification analysis of photosynthetic proteins of *M. afer* under different culture conditions. **(A)** Western-blot analyses of D1 protein under different culture conditions. Thylakoid membrane proteins were separated by 12% SDS-PAGE and subsequently probed with D1 antibody. Thylakoid samples were loaded on the basis of equivalent total protein content (15 μg) in each lane. 1/4 CT and 1/2 CT: a fourfold and twofold dilution of extract of control group. **(B)** The amounts of D1 in *M. afer* harvested from different culture conditions were calculated based on the gray analysis of western-blot by Image J software. **(C)** Isolated thylakoid membrane proteins under different culture conditions on SDS-PAGE. Thylakoid samples were loaded on the basis of equivalent total protein content (15 μg) in each lane. **(D)** Semi-quantification analysis of two target bands (27 and 50 kDa) according to their gray scale. Values are means ± SE (*n* = 3). The insert table showed the protein predicted by LC–MS/MS. Asterisks indicate statistically significant differences compared with the control group (^∗^*p* < 0.05; ANOVA).

The cells of two experimental groups (0.1 NAA+50 TP and 0.1 NAA+500 TP) which had the highest lipid yield were harvested for further quantification analysis of photosynthetic proteins. As the core photosynthetic apparatus of PSII, the abundance of chloroplast photosynthetic protein D1 could represent the amount of functional PSII in algal cells (Schnettger et al., 1994). According to the western-blot result, D1 content in the cells that cultured with 0.1 mg/L NAA and 500 mg/L TP supplement was 33.3% higher than control (**Figures 5A,B**). Dilutions of control group were used to proving the accuracy of western-blotting process. Combined with the ROS level results, it is speculated that the cells with higher TP supplement were suffered less oxidant damage on the PSII.

In order to get more clues about how TP induced the high robust of PSII to response to high ROS stress, thylakoid membrane proteins were analyzed by SDS-PAGE. Four bands at 24, 25, 27, and 50 kDa were chosen for further composition and semi-quantification analysis (Supplementary Figure 1). Based on our western-blotting experience of D1 protein (two bands with close dimensions at 25 kDa), bands at 24 and 25 kDa are D1 and pD1 proteins (precursor D1 protein), respectively. Two bands at 50 and 27 kDa exhibit significant differences between the treatment samples and control by the gray scale analysis of blue Coomassie-stained gel (**Figure 5D**). Further LC–MS/MS analysis predicted these are the protein bands of CP43 (PSBC_CHLEU) and D2 (PSBD_BIGNA), respectively (**Figure 5D** and Supplementary Table 6). With TP supplement, the amount of D2 protein was 25.6% higher than control, while the amount of CP43 was 84.2% lower than control (**Figures 5C,D**).

## Discussion and Conclusion

Auxins play a particular role in higher plant development by affecting several physiological processes (Perrot-Rechenmann, 2010). Nowadays, the presence of auxins in algal lineages were also demonstrated, and parts of their effects on cell growth and development were uncovered (Lu and Xu, 2015). Microalgae might have similar auxin signal and response system of plants (Lau et al., 2009). Auxin signaling, like transport inhibitor response1-auxin signaling F-box protein (TIR1-AFB), auxin response factor (ARF) and auxin-indole-3-acetic acid proteins (AUX-IAA), has been studied (Gray et al., 2001; Tiwari et al., 2003; Parry et al., 2009). Evidence for phytohormone function in microalgae is rare but beginning to accumulate. For instance, auxin was demonstrated to induce cell division in the unicellular *desmid Micrasterias thomasiana* (*Charophyta*) and *Chlorella pyrenoidosa* (*Chlorophyta*) (Wood and Berliner, 1979; Vance, 1987). Two AUX-IAAs were presented in *C. reinhardtii* (Palenik et al., 2007). As exogenous synthetic auxin, effects of NAA on the growth and lipid accumulation of *M. afer* were investigated in this study. According to the orthogonal experiments, the cell growth of *M. afer* would be promoted by NAA at the concentration below 1 mg/L, further it was confirmed by the single factor analysis with 0.1 mg/L NAA treatment. This concentration brought the most significant effect to the growth of *M. afer* at the 2nd day after supplement, which may be due to the regulatory effect of NAA in cell division (Palenik et al., 2007). Furthermore, NAA was stable in the culture system, and had a better growth promoting effect when combined with TP supplement in the later growth stage of *M. afer*.

The growth and lipid accumulation of *M. afer* were promoted with antioxidant supplement in our study. TP includes catechins, theaflavins, tannins, and flavonoids. Catechins are the main component of TP (60–80%), and are mainly composed of EGCG (50%), epicatechin-3-gallate (ECG, 20%), epigallocatechin (EGC, 20%), epicatechin (EC), catechin, and gallocatechin (GC) (Lin et al., 1996; Frei and Higdon, 2003). TP and EGCG were investigated as antioxidant additives in this study, and showed similar effects on *M. afer* growth. Due to the consideration of large-scale production application, TP would be more economical and it was selected for further investigation in this work. With the addition of 0.1 mg/L NAA and 500 mg/L TP, the final lipid yield of *M. afer* was 2.15 g/L, which was 39.6% higher than control. The extra fee for additives addition mainly comes from TP, whose price is 60–80 CNY per kilogram for large-scale food grade raw material procurement. It only additional accounted for 15–20 CNY to harvest 1 kg of algae oil with high NA content (5%).

**FIGURE 6:**
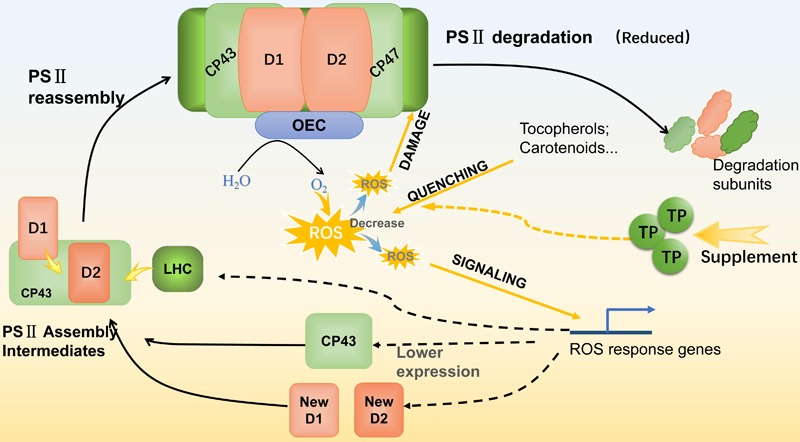
Schematic diagram of the PSII repair cycle under the supplement of TP. With TP supplement, ROS quenching was enhanced and lead to the decrease of ROS for damage and signaling processes. As the result of less ROS damaging, PSII degradation was reduced and performed as the increasing of D1 and D2 content. For ROS signaling, a less amount of CP43 gives the hint that PSII re-assembly process was decelerated. Colored squares are core protein components (D1, D2, CP43, CP47, LHC, and OEC) of PSII. LHC, light harvesting complex; OEC, oxygen evolving complex.

With the supplement of NAA and TP, the variation of intracellular ROS level was observed, the changes of photosynthetic efficiency were also significant. As a photosynthetic organism, algae converted light energy into chemical energy for life activities or stored in the form of biochemical compounds. However, the excess light energy could lead to excess ROS accumulation and photo-oxidative damage to the photosynthetic apparatus (Mullineaux and Karpinski, 2002). Photosynthetic organisms need to alleviate the detrimental effects of high ROS stress, especially under stressful conditions (Kolber et al., 1988). In this work, there was an obvious linear relationship between the photosynthetic efficiency and intracellular ROS level. As expected, *M. afer* cultured with TP was detected a lower ROS level, MDA content, and CAT activity than the control. These indicated that the cells of *M. afer* cultured with TP supplement were under lower oxidant stress (Zhang et al., 2013). The variation of CAT activity was due to the ROS quenching effort of TP, lower activity of CAT is enough for the ROS neutralization. Further analysis of thylakoid membrane proteins by SDS-PAGE and western-blot analysis, the amounts of D1 and D2 proteins in cells cultured with TP were higher than the control, while the amounts of CP43 protein decreased 84.2% compared with the control. We speculated that TP possesses the function of slowing down the damage of PSII by scavenging excess intracellular ROS and resulting the higher amount of D1 and D2 in the cells. Photo-oxidative damages of PS II are frequently generated by breaking its reaction center and damaging the thylakoid membranes. Organisms maintain the photosynthetic ability through an intricate repair mechanism involving degradation of the damaged D1 reaction center protein and re-assembly by fresh core skeleton proteins into the photosynthetic system (Andersson and Aro, 2001). Under stress conditions, the rate of photo-oxidative damage exceeds the capacity of self-repair, which leads to a decrease of D1 and D2 abundance in photosynthetic system (Krieger-Liszkay et al., 2008). The higher amounts of D1 and D2 proteins might indicated a less degradation of PSII (Erickson et al., 2015). Moreover, instead of the previously known function of CP43, which acted as one of the core PSII antenna proteins. It was also demonstrated the Loop E of CP43 played a crucial role in the assembly of the water oxidizing center during PSII biosynthesis. The structural dynamics of the luminal domain of CP43 determined its role in the assembly of functional PSII centers (Liu et al., 2013). The reduction of CP43 could be associated with the retard of PSII assembly. Based on these, we hypothesis that the ROS level could play a role on the expression of CP43. This was confirmed by our results of ROS analysis. When cells were supplemented with TP, they had a lower ROS level, which lead to a less PSII degradation. Furthermore, the lower ROS level would cause the down-regulation of CP43 protein and less re-assembly of PSII. The regulation pathway from ROS to CP43 is interesting and will be analyzed by future research. In summary, supplement of TP enhanced ROS quenching, therefore the amount of ROS targeted to PSII damage and PSII repair signaling was decreased, which leaded to a less PSII damage (**Figure 6**). As a result, TP could reduce the damage of PSII by scavenging intracellular excess ROS at later growth stage. The cells supplied with NAA and TP could get higher growth rate, the energy and reducing power could accumulated in the form of fatty acids.

## Author Contributions

YF: conceived and designed the experiments. FX, YF, and G-RH: performed the experiments. YF, FM, and JS: analyzed the data and discussed the results. YF, FX, F-LL, and GY: wrote the paper.

## Conflict of Interest Statement

The authors declare that the research was conducted in the absence of any commercial or financial relationships that could be construed as a potential conflict of interest.
