# Supplementary material for: Naphthylacetic Acid and Tea Polyphenol Application Promote Biomass and Lipid Production of Nervonic Acid-Producing Microalgae
Source: Front Plant Sci. 2018 Apr 17;9:506. doi: 10.3389/fpls.2018.00506 (PMC5920212; doi:10.3389/fpls.2018.00506)
Supplement: Supplementary file 1 [file Table_1.docx]

**Supplementary Figure 1.** SDS-PAGE of isolated thylakoid membrane proteins under different culture conditions.


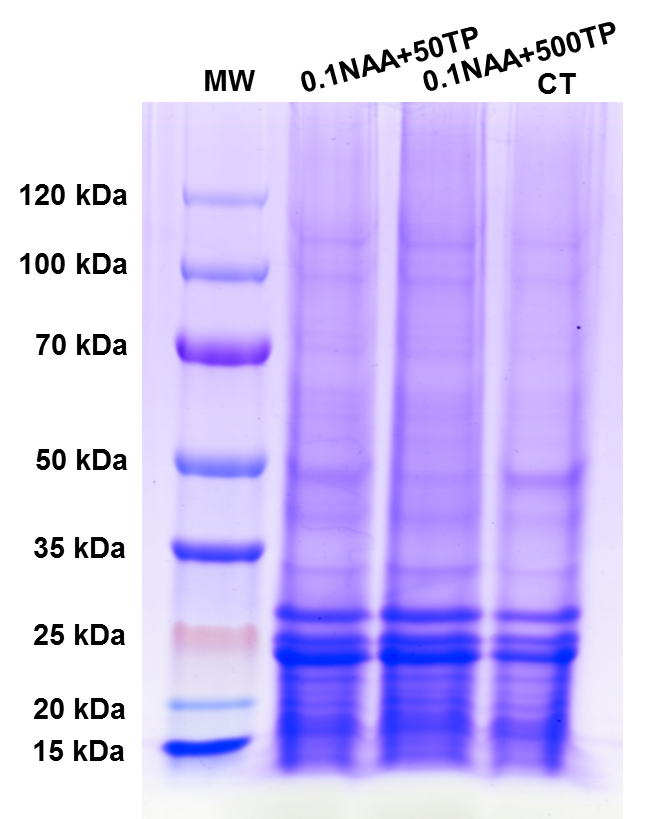


Supplementary Table 1. Orthogonal experiments scheme and results

| Code | NAA (mg/L) | Antioxidants (mg/L) | NULL | Light intensity | B×C | OD750 | YⅡ | Nile red fluorescence |
| --- | --- | --- | --- | --- | --- | --- | --- | --- |
| 1 | A_1_ | B_1_ | 1 | C_1_ | 1 | 8.05 | 0.181 | 371.56 |
| 2 | A_1_ | B_2_ | 2 | C_2_ | 2 | 8.03 | 0.184 | 324.26 |
| 3 | A_1_ | B_3_ | 3 | C_3_ | 3 | 8.95 | 0.147 | 455.30 |
| 4 | A_1_ | B_4_ | 4 | C_4_ | 4 | 9.25 | 0.168 | 419.05 |
| 5 | A_2_ | B_1_ | 2 | C_3_ | 4 | 9.14 | 0.136 | 456.12 |
| 6 | A_2_ | B_2_ | 1 | C_4_ | 3 | 9.04 | 0.134 | 566.93 |
| 7 | A_2_ | B_3_ | 4 | C_1_ | 2 | 8.17 | 0.271 | 330.81 |
| 8 | A_2_ | B_4_ | 3 | C_2_ | 1 | 8.23 | 0.295 | 388.77 |
| 9 | A_3_ | B_1_ | 3 | C_4_ | 2 | 8.98 | 0.091 | 549.10 |
| 10 | A_3_ | B_2_ | 4 | C_3_ | 1 | 9.06 | 0.105 | 488.94 |
| 11 | A_3_ | B_3_ | 1 | C_2_ | 4 | 7.92 | 0.191 | 400.80 |
| 12 | A_3_ | B_4_ | 2 | C_1_ | 3 | 8.06 | 0.26 | 338.43 |
| 13 | A_4_ | B_1_ | 4 | C_2_ | 3 | 7.59 | 0.166 | 317.63 |
| 14 | A_4_ | B_2_ | 3 | C_1_ | 4 | 7.69 | 0.151 | 311.56 |
| 15 | A_4_ | B_3_ | 2 | C_4_ | 1 | 9.12 | 0.119 | 391.21 |
| 16 | A_4_ | B_4_ | 1 | C_3_ | 2 | 8.94 | 0.121 | 420.12 |

Supplementary Table 2. Variance analysis to OD_750_

| Source | Type Ⅲ Sum of Square | df | Mean Square | F | Sig. |
| --- | --- | --- | --- | --- | --- |
| A (NAA) | 0.210 | 3 | 0.070 | 5.978 | 0.088 |
| B (Antioxidants) | 0.083 | 3 | 0.028 | 2.380 | 0.248 |
| C (Light intensity) | 4.790 | 3 | 1.597 | 136.578 | 0.001 |
| B×C | 0.086 | 3 | 0.029 | 2.448 | 0.241 |
| Error | 0.035 | 3 | 0.012 |  |  |
| Total | 1164.948 | 16 |  |  |  |
| Corrected Total | 5.205 | 15 |  |  |  |

Dependent Variable: OD_750_

Supplementary Table 3. Variance analysis to YⅡ

| Source | Type Ⅲ Sum of Square | df | Mean Square | F | Sig. |
| --- | --- | --- | --- | --- | --- |
| NAA (A) | 0.010 | 3 | 0.003 | 9.925 | 0.046 |
| Antioxidant (B) | 0.013 | 3 | 0.004 | 12.646 | 0.033 |
| Light intensity (C) | 0.029 | 3 | 0.010 | 28.216 | 0.011 |
| B×C | 0.001 | 3 | 0.000 | 0.601 | 0.657 |
| Error | 0.001 | 3 | 0.000 |  |  |
| Total | 0.516 | 16 |  |  |  |
| Corrected Total | 0.054 | 15 |  |  |  |

Dependent Variable: YⅡ

Supplementary Table 4. Variance analysis to Nile red fluorescence intensity

| Source | Type Ⅲ Sum of Square | df | Mean Square | F | Sig. |
| --- | --- | --- | --- | --- | --- |
| NAA (A) | 18457.1 | 3 | 6152.3 | 1.75 | 0.328 |
| Antioxidant (B) | 3666.6 | 3 | 1222.2 | 0.348 | 0.795 |
| Light intensity (C) | 60136.1 | 3 | 20045.3 | 5.71 | 0.093 |
| B×C | 1062.5 | 3 | 354.1 | 0.101 | 0.954 |
| Error | 10527.8 | 3 | 3509.2 |  |  |
| Total | 2759387.9 | 16 |  |  |  |
| Corrected Total | 93850.1 | 15 |  |  |  |

Dependent Variable: Nile red fluorescence intensity

Supplementary Table 5. The compositions of fatty acids in different conditions

| Fatty acids (%) | CT | 0.1NAA | 10NAA | 50TP | 500TP | 0.1NAA+50TP | 0.1NAA+500TP | 10NAA+50TP | 10NAA+500TP |
| --- | --- | --- | --- | --- | --- | --- | --- | --- | --- |
| C16:0 | 22.67±0.6 | 24.05±0.8 | 21.53±0.8 | 23.51±0.7 | 25.02±1.2 | 24.21±0.4 | 22.17±0.8 | 23.46±0.7 | 23.17±1.3 |
| C16:1 | 5.94±0.44 | 5.86±1.1 | 6.37±1.4 | 5.47±0.74 | 4.48±0.94 | 5.81±0.63 | 5.96±1.2 | 4.81±0.59 | 5.91±0.72 |
| C16:2 | 2.85±0.51 | 2.35±0.49 | 2.78±0.73 | 2.39±0.24 | 1.94±0.38 | 2.28±0.51 | 2.02±0.43 | 2.81±0.62 | 2.70±0.89 |
| C16:3 | 1.43±0.26 | 1.52±0.35 | 1.35±0.21 | 1.38±0.36 | 1.64±0.45 | 1.48±0.27 | 1.47±0.49 | 1.51±0.47 | 1.15±0.33 |
| C18:0 | 3.58±0.49 | 3.48±0.74 | 3.44±0.59 | 3.57±0.85 | 3.52±0.51 | 3.92±0.91 | 3.33±0.87 | 3.72±0.59 | 3.31±0.62 |
| C18:1 | 46.11±2.1 | 46.13±1.1 | 47.69±0.5 | 47.29±1.1 | 47.25±1.6 | 43.55±0.9 | 47.30±2.1 | 44.68±0.8 | 46.30±0.9 |
| C18:2 | 6.67±0.87 | 6.08±0.74 | 6.15±0.84 | 5.96±0.47 | 5.11±0.38 | 5.89±0.84 | 5.55±0.48 | 7.22±0.71 | 6.36±0.34 |
| C18:3 | 6.88±0.71 | 6.82±0.38 | 7.08±0.64 | 6.93±0.82 | 7.01±1.02 | 9.05±0.87 | 8.40±0.95 | 7.89±0.54 | 7.16±0.64 |
| C24:1 | 3.87±0.24 | 3.71±0.18 | 3.61±0.35 | 3.50±0.12 | 3.67±0.28 | 3.82±0.08 | 3.80±0.14 | 3.92±0.21 | 3.93±0.34 |

Supplementary Table 6. Identification of two target bands (27 kDa and 50 kDa) by LC-MS/MS. The tables were shown in the supplementary EXCEL table.
